# Developing and testing a case-management intervention to support the return to work of health care workers with common mental health disorders

**DOI:** 10.1093/pubmed/fdac055

**Published:** 2022-05-30

**Authors:** V Parsons, D Juszczyk, G Gilworth, G Ntani, M Henderson, J Smedley, P McCrone, S L Hatch, R Shannon, D Coggon, M Molokhia, A Griffiths, K Walker-Bone, I Madan

**Affiliations:** Occupational Health Service, Guy’s & St Thomas’ NHS Foundation Trust, London SE1 7NJ, UK; Faculty of Life Sciences & Medicine, King’s College London, London SE1 9NH, UK; Occupational Health Service, Guy’s & St Thomas’ NHS Foundation Trust, London SE1 7NJ, UK; Occupational Health Service, Guy’s & St Thomas’ NHS Foundation Trust, London SE1 7NJ, UK; MRC Lifecourse Epidemiology Centre, University of Southampton, Southampton SO16 6YD, UK; MRC Versus Arthritis Centre for Musculoskeletal Health and Work, University of Southampton, Southampton SO16 6YD, UK; Leeds Institute of Health Sciences, University of Leeds, Leeds LS2 9JT, UK; Occupational Health, University Hospital Southampton NHS Foundation Trust, Southampton SO16 6YD, UK; King’s Health Economics, King’s College London, London SE1 9NH, UK; Faculty of Education, Health & Human Sciences School of Health Sciences University of Greenwich, King’s College London, London SE19NH, UK; Department of Psychological Medicine, Institute of Psychiatry, Psychology & Neuroscience, Kings College London, London SE5 8AF, UK; School of Health Sciences, University of Southampton, Southampton SO14 0YN, UK; MRC Lifecourse Epidemiology Centre, University of Southampton, Southampton SO16 6YD, UK; Department of Population Health Sciences, School of Life Course and Population Sciences, Population Health Sciences, King’s College London, London SE1 1UL, UK; Mental Health & Neurosciences, School of Medicine, Institute of Mental Health, University of Nottingham, Nottingham NG7 2UH(UK), UK; MRC Lifecourse Epidemiology Centre, University of Southampton, Southampton SO16 6YD, UK; MRC Versus Arthritis Centre for Musculoskeletal Health and Work, University of Southampton, Southampton SO16 6YD, UK; Occupational Health Service, Guy’s & St Thomas’ NHS Foundation Trust, London SE1 7NJ, UK; Faculty of Life Sciences & Medicine, King’s College London, London SE1 9NH, UK

**Keywords:** case-management, health care workers, interventions, mental health, occupational health, sickness absence

## Abstract

**Background:**

To assess the feasibility and acceptability of conducting a trial of the clinical effectiveness and cost-effectiveness of a new case-management intervention to facilitate the return to work of health care workers, on sick leave, having a common mental disorder (CMD).

**Methods:**

A mixed methods feasibility study.

**Results:**

Systematic review examined 40 articles and 2 guidelines. Forty-nine National Health Service Occupational Health (OH) providers completed a usual care survey. We trained six OH nurses as case managers and established six recruitment sites. Forty-two out of 1938 staff on sick leave with a CMD were screened for eligibility, and 24 participants were recruited. Out of them, 94% were female. Eleven participants received the intervention and 13 received usual care. Engagement with most intervention components was excellent. Return-to-work self-efficacy improved more in the intervention group than in the usual care group. Qualitative feedback showed the intervention was acceptable.

**Conclusions:**

The intervention was acceptable, feasible and low cost to deliver, but it was not considered feasible to recommend a large-scale effectiveness trial unless an effective method could be devised to improve the early OH referral of staff sick with CMD. Alternatively, the intervention could be trialled as a new stand-alone OH intervention initiated at the time of usual OH referral.

## Introduction

High rates of sickness absence due to common mental disorders (CMD) remain a serious occupational burden for staff and employers,^1^^,^^2^ despite a commitment by many organizations to promote a ‘mentally healthy’ workplace culture, and the increasing proliferation of workplace health and well-being initiatives. The National Health Service (NHS) in England employs ~1 million staff.^3^ Estimates suggest over a quarter of NHS staff sickness absence is due to mental ill health.^4–7^ While over 75% of staff on sick leave with a mental disorder eventually return to work (RTW),^8^ staff absent for ≥6 months have a <50% chance of returning to employment.^9^ In addition to economic benefits from reducing sickness absence, there is a proven association between staff well-being and enhanced patient care.^7^^,^^10^

The literature on workplace interventions to improve work participation for people on sick leave highlights poor quality evidence on the effectiveness of such interventions in staff with CMD.^11^^,^^12^ Reviews suggest that occupational health (OH) interventions that comprise clinical and workplace multi-components to support RTW following sickness absence due to CMD can be effective,^12–15^ however, none have been evaluated with health care staff. Evidence on the optimal timing for the delivery of RTW interventions to reduce the period of sickness absence remains uncertain; current recommendations vary between 1 day and 12 weeks.^15–20^

Case-management approaches grounded in a biopsychosocial framework are found to be cost-effective in supporting health care staff to RTW following sickness absence for non-mental health reasons.^19^^,^^20^ Here, we aim to develop and assess the acceptability of a bespoke OH intervention to reduce the sickness absence period for NHS workers off work with CMDs and to determine the feasibility of conducting a trial comparing the clinical effectiveness and cost-effectiveness of the intervention compared to care as usual.

## Methods

A mixed methods study design comprising four work packages (WPs).

In WP1, we updated a systematic review carried out by Pomaki *et al*.^21^ This work is described elsewhere.^22^ In WP1, we also undertook a cross-sectional survey of NHS OH departments in the UK to establish usual care in the management of NHS staff taking sick leave with a CMD.^23^

In WP2, we mapped the published evidence and expert and stakeholder feedback onto the new case-management intervention, which was designed to be delivered by experienced OH nurses following bespoke training. It comprised: comprehensive occupational and mental state assessment at the first and follow-up consultations; identification of facilitators and obstacles to RTW; problem identification and problem-solving focussed on achieving RTW (partial or full); local peer-support networking; optimization and encouraging adherence to clinical treatment; provision of resource material for participants and line managers to support RTW; signposting to complementary follow-up support services and personalized goal setting and action planning. The intervention also included a tailored, written RTW plan outlining personalized workplace adjustments and modifications to facilitate RTW, which was developed following discussion between participants and their manager and was shared with the participants’ health care professionals. Regular timed reviews (2–4 weeks) monitored the progress. Outcome measures were: self-reported change in anxiety/depression; change in use of psychiatric medication; extent of RTW (early, part, full and sustained); change in health-related quality of life; relapse rates and adverse events (self-harm/suicide). Validated tools and questionnaires were used to assess outcomes, including e.g. EQ-5D-5L, Generalised Anxiety Disorder scale (GAD-7), the Patient Health Questionnaire tool (PHQ-9) and Whooley questions, WHODAS, alcohol use tool, Work and Social Adjustment Scale, RTW-Self-Efficacy scale and Client Service Receipt Inventory (CSRI) which measures utilization of health care and associated services. For the set of questions assessing anxiety and depression, a final score was produced for each condition across three time periods (baseline, 3 months and 6 months) and a categorical variable for each condition was produced in keeping with standard practice.^24^^,^^25^ Cost-effectiveness of the intervention was assessed. (Note: Because this was a feasibility study, we did not focus on the costs of health services but rather their use, and we did not conduct a cost-effectiveness analysis. This would need to be done in a full trial).

In WP3, we delivered a 2-day case manager training workshop for experienced (minimum 2 years) OH nurses (case managers). The training was delivered face-to-face by co-investigators experienced in delivering educational training to clinicians. The workshop covered study procedures for data collection; clinical assessment of CMD; risk assessment; identifying and challenging unhelpful beliefs associated with RTW; problem identification and problem-solving which emphasized personalized goal-setting taking into account individual’s strengths and capacity; promotion of engagement (theory and practice using basic motivational interviewing techniques); use of clinical proformas and RTW planning.

Following this, we conducted a feasibility study. Staff were eligible for the study if they had been off sick for between 7 and 90 days with a CMD. We excluded staff with psychosis, bipolar disorder, substance misuse disorders or dementia and those under investigation for misconduct or in the disciplinary process. Case file audits were conducted to evaluate intervention fidelity.

In WP 4, we manualized the intervention, taking into consideration the results from the feasibility study and compiled recommendations for the future development and design of a main trial. The feasibility study included a qualitative process evaluation comprising recorded interviews with case managers and participants receiving case-management support and stakeholder focus groups.

### Analysis

Characteristics of eligible participants were recorded at baseline and were summarized through means, medians, standard deviations (SDs) and inter-quartile ranges (IQRs). The qualitative data in the transcripts from the interviews and focus group were analysed using a thematic analysis.^26^

## Results

### WP 1 systematic review and survey of usual care

Full descriptions are published elsewhere.^22^^,^^23^^,^^27^

### WP 2 development of the case-management intervention, case manager training workshop and data collection tools

**Fig. 1:**
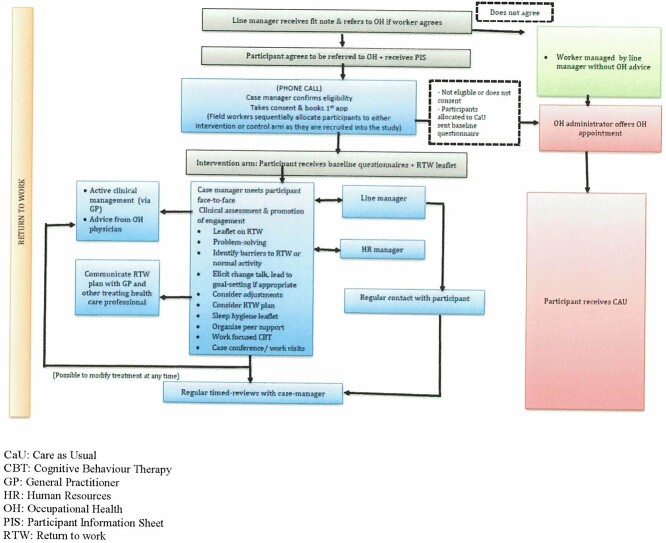
Manualized model of the integrated case-management intervention (including recruitment and care as usual pathway).

We developed a work-focused case-management intervention (Fig. 1) and developed a 2-day case manager training workshop as described above. We also produced and tested the acceptability and utility of data collection tools to measure outcomes and assess adherence to, and acceptability of, the intervention and study processes.

### WP 3 Case manager training workshop

The workshop was facilitated by investigators representing different clinical backgrounds (occupational medicine, psychiatry and psychology), each with experience delivering clinician training. Six experienced OH nurses from four NHS Trusts completed the case manager training. We conducted pre- and post-workshop evaluations which showed an increase in knowledge, confidence and skill acquisition specific to the delivery of the new intervention. Although, we noted knowledge diminished for several questions at post-training evaluation (Table 1).

**Table 1:** Pre- and post-case-management training knowledge assessment scores based on overall group responses

Question No.	Pre-knowledge	Post-knowledge	CHANGE, +/−
Question 1: The presence of prominent physical symptoms (such as pain and fatigue) in a depressed person means they probably also have an underlying physical health problem	5/6 correct	4/6 correct	Decrease
Question 2: Discussing the topic of suicide will increase the risk of a patient harming themselves in the future	6/6 correct	6/6 correct	All correct
Question 3: Women are less likely than men to commit suicide	6/6 correct	4/6 correct	Decrease
Question 4: The majority of those who kill themselves have complained of psychological distress to their GP in the preceding month	5/6 correct	5/6 correct	No change
Question 5: Of patients who have had one episode of major depression, 50–85% will go on to have a second episode	5/6 correct	3/6 correct	Decrease
Question 6: In panic disorder, the timing of panic attacks is usually unpredictable	4/6 correct	5/6 correct	Increase
Question 7: The likelihood of a worker returning to work after an episode of depression is strongly determined by the severity of their depression	4/6 correct	4/6 correct	No change
Question 8: The worker’s expectation of RTW is strongly predictive of when and if they will RTW	6/6 correct	6/6 correct	All correct
Multiple choice question.	Group answers	Group answers	CHANGE, +/−
Question 9: The stages of problem-solving include			
Question 9a: setting a problem list	2/6 correct	4/6 correct	Increase
Question 9b: reviewing past successes and failures	4/6 correct	4/6 correct	No change
Question 9c: examining the patient/therapist relationship	5/6 correct	5/6 correct	No change
Question 9d: setting achievable goals	5/6 correct	5/6 correct	No change
Question 9e: defining problems precisely	2/6 correct	2/6 correct	No change
Question 10: Achievable goals in problem-solving should be			
Question 10a: chosen by the therapist	6/6 correct	5/6 correct	Decrease
Question 10b: achieved before the next treatment session	6/6 correct	5/6 correct	Decrease
Question 10c: achieved within a defined time span	1/6 correct	4/6 correct	Increase
Question 10d: related to problems chosen	4/6 correct	6/6 correct	Increase
Question 10e: linked to relationship difficulties	6/6 correct	6/6 correct	All correct
Question 11: Problem-solving treatment is a proven treatment for			
Question 11a: depressive disorders	2/6 correct	6/6 correct	Increase
Question 11b: simple phobias	5/6 correct	4/6 correct	Decrease
Question 11c: mania	6/6 correct	6/6 correct	All correct
Question 11d: adolescent depression	6/6 correct	6/6 correct	All correct
Question 11e: anxiety disorders in primary care	1/6 correct	3/6 correct	Increase
Question 12: Problem-solving treatment is most effective when			
Question 12a: combined with antidepressant medication	2/6 correct	1/5 (one didn’t answer)	No change
Question 12b: when delivered by community nurses	6/6 correct	5/5 (one didn’t answer)	All correct
Question 12c: when delivered over ten sessions	6/6 correct	5/5 (one didn’t answer)	All correct
Question 12d: patients have many psychosocial problems	6/6 correct	5/5 (one didn’t answer)	All correct
Question 12e: patients have a chronic illness	5/6 correct	5/5 (one didn’t answer)	All correct
Question 13: SMART goals are			
Question 13a: simple	3/6 correct	3/6 correct	No change
Question 13b: modest	5/6 correct	4/6 correct	Decrease
Question 13c: achievable	4/6 correct	6/6 correct	Increase
Question 13d: resisted	5/6 correct	5/6 correct	No change
Question 13e: timed	2/6 correct	5/6 correct	Increase

Overall knowledge decreased/increased from pre- versus post-training: decrease = 7, increase = 8, no change = 13.

### The feasibility study

**Fig. 2:**
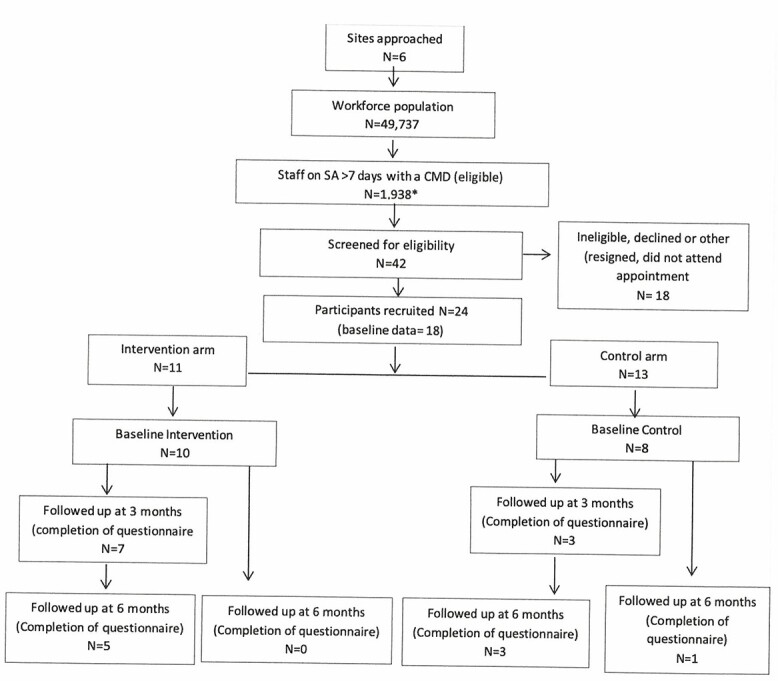
Consolidated Standards of Reporting Trial flow chart.

Five NHS Trusts in England (UK) participated in the feasibility study. Workforce intelligence data, showed that ~49 737 staff were employed across these trusts, and of these, 1938 (3.9%) staff were recorded as being on sick leave for >7 days with a CMD. Forty-two staff who were off sick were subsequently screened by OH departments for eligibility upon receipt of OH referrals. From these, 24 (57%) staff met the inclusion criteria and were consented into the study. Eleven of the 24 participants (46%) received the case-management intervention and 13 (54%) received usual care (Fig. 2). We collected baseline data from 18 (75%) participants.

As shown in Table 2, 94% of participants were female with a mean age of 43 years. The highest proportion of participants were categorized as nursing, midwifery and health visiting staff. On average, participants worked >30 hours per week and the majority reported working day shifts only.

**Table 2:** Baseline characteristics of participants

	Total, N = 18
Age (mean (SD)/median (IQR))	42.8 (12.9)/42 (34–55)
Females (*N* (%))	17 (94)
Main job role (*N* (%))	
Administration and estate staff	3 (17)
Health care assistants and other support staff	3 (17)
Health care scientists	1 (6)
Nursing, midwifery and health visiting staff	7 (39)
Nursing, midwifery and health visiting learners	1 (6)
Other	1 (6)
Scientific, therapeutic and technical staff	2 (11)
Contracted hours (mean (SD)/median (IQR))	31.5 (7.8)/36.8 (26-37.5)
Day shifts only	14 (78)
Day and night shifts	4 (22)
Frequency of alcohol use	Never: 5 (28) Less than monthly: 4 (22) Monthly: 4 (22) Weekly: 5 (28) Daily: 0
Prior history of mental illness	No: 4 (22) Yes: 14 (78)
If yes, sickness absence due to mental illness	No: 6 (43) Yes: 6 (43) Missing: 2 (14)
Treatment for mental illness (medicines or talking therapy)	No: 2 (14) Yes: 11 (79) Missing: 1 (7)
Other health condition (non-mental health)	No: 4 (22) Yes: 14 (78)
If yes, what	Respiratory: 4 (29) Cardiac: 1 (7) Musculoskeletal: 1 (7) Neurological: 1 (7) Other: 7 (50)
If other, specify	Gallstones/gall bladder removal Diabetic symptoms Eye sight and hearing Gastrointestinal Irritable bowel syndrome (possible inflammatory bowel disease) Sinusitis Bowel disease
Sickness absence due to health condition (not metal health)	No: 11 (61) Yes: 4 (22) Missing: 3 (17)

We assessed the impact of CMDs on work and occupational functioning at baseline only and found that the median number of days off sick was 3.8 for participants in the 12 months before entering the study. Over a third (*n* = 7; 39%) of participants expected their current period of sick leave would be <4 weeks, whereas over half (*n* = 11; 61%) expected their absence to last >4 weeks. Over half (*n* = 13; 72%) of participants rated their level of job satisfaction as ‘moderately satisfied’ to ‘extremely satisfied’.

### Distribution of outcome measures

Over half of participants (*n* = 13; 72%) self-reported moderately severe to severe anxiety at baseline, which improved as participants progressed through the study (Table 3). This was also reflected in the measure of depression severity, with over half of participants (*n* = 11; 61%) reporting moderately severe to severe depression at baseline, which improved at follow-up time-points, with milder symptoms reported by participants in the intervention group.

**Table 3:** Distribution of outcome measures

	Baseline, N = 18 (%)	3 months, N = 11 (%)	6 months, N = 10 (%)	6 months care as usual, N = 5 (%)	6 months intervention, N = 5 (%)
Anxiety (continuous score)	Mean (SD): 14.8 (4.4) Median (IQR): 15.5 (10.0–18.0)	Mean (SD): 6.6 (6.3) Median (IQR): 4.0 (3.0–9.0)	Mean (SD): 7.2 (6.7) Median (IQR): 6.5 (0–12.0)	Mean (SD): 8.0 (9.0) Median (IQR): 6.0 (0–12.8)	Mean (SD): 6.4 (4.3) Median (IQR): 7.0 (6.0–7.0)
Anxiety (grouped)					
Mild	0	6 (55)	3 (30)	2 (40)	1 (20)
Moderate	5 (28)	3 (27)	4 (40)	1 (20)	3 (60)
Moderately severe	4 (22)	1(9)	2 (20)	1 (20)	1 (20)
Severe	9 (50)	1 (9)	1 (10)	1 (20)	0
Depression (continuous score)	Mean (SD): 16.4 (5.5) Median (IQR): 18.0 (12.4–21.0)	Mean (SD): 9.5 (8.3) Median (IQR): 9.0 (3.0–14.0)	Mean (SD): 9.5 (8.5) Median (IQR): 7.0 (4.0–14.0)	Mean (SD): 9.8 (12.0) Median (IQR): 4.0 (0–19.0)	Mean (SD): 9.2 (4.3) Median (IQR): 9.0 (5.0–13.0)
Depression (grouped)					
None	1(6)	4 (36)	3 (30)	3 (60)	0
Mild	0	2 (18)	3 (30)	0	3 (60)
Moderate	6 (33)	3 (27)	2 (20)	0	2 (40)
Moderately severe	5 (28)	0	1 (10)	1 (20)	0
Severe	6 (33)	2 (18)	1 (10)	1 (20)	0

GAD-7 Anxiety severity scoring: 0–4 (minimal), 5–9 (mild), 10–14 (moderate), 15–20 (severe). PHQ- 9 Depression severity scoring: 0–4 (none/minimal), 5–9 (mild), 10–14 (moderate), 15–19 (moderately severe), 20–27 (severe).

We found longer consultation times and more appointments for participants receiving the intervention compared to usual care, i.e. (mean) 85 minutes compared to 45 minutes (at the first appointments) and 71 minutes compared to 45 minutes at the second and subsequent appointment. Participants in the intervention arm had access to up to six follow-up appointments with their case manager compared to usual care participants who had up to three follow-up appointments. In addition, we found use of antidepressants increased slightly during the study period in both arms. In terms of acceptability, the majority of participants who accessed and used key components of the intervention rated them favourably.

**Fig. 3:**
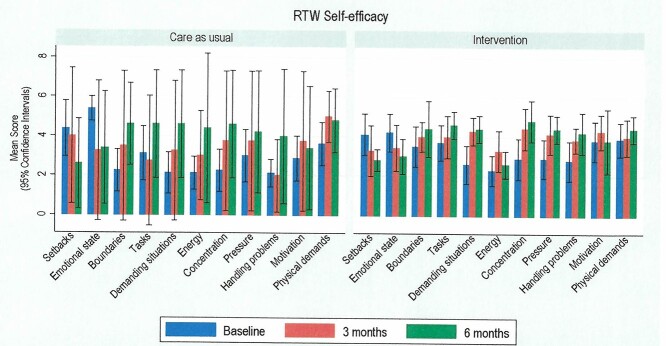
Self-report measures of participants self-efficacy associated with RTW across different domains.

In Fig. 3, the results showed that there was a general positive trend towards improvements in RTW self-efficacy from baseline to 3 months and 6 months for participants in the intervention arm when compared to participants in the CAU arm, although the overall numbers were small.

We have not reported on participants’ perceived capacity to function in the workplace (i.e. their self-assessed ‘workability’) since, in some categories, the numbers were very small resulting in very wide confidence intervals, meaning it is difficult to draw reliable inferences from the data. Furthermore, we have not included outcome measure data on RTW, given the very poor level of agreement observed between our two data sources (i.e. self-report versus case managers). This additional data are reported elsewhere.^27^

We assessed adherence to the case-management intervention via an audit of the clinical notes. While all participants engaged in the problem-solving components, written RTW plans were produced and sleep hygiene and RTW leaflets were provided to all participants and their line managers, and we found no evidence that participants engaged in the peer-support component. No case conferencing or workplace visits were arranged.

### Health economics

While the CSRI was reasonably well reported, with the exception of GP contact, the majority of health care services (psychiatrist/psychologist/social worker, Fit for Work service) were not accessed by participants. This low level of the engagement suggests that a shortened version of the tool would be suitable for use in a full trial. The EQ-5D-5I tool was completed satisfactorily and, from this, we found that the majority of participants reported no significant impairment with mobility and self-care at baseline. The most noticeable variability in both arms at baseline and follow-up regarded usual activities, pain/discomfort and anxiety/depression domains. The intervention costs consisted of fixed elements for training and materials, i.e. £10 788 for case manager training, £5025 for resources and materials and £954 for intervention delivery. For the 11 recipients who received the intervention, the therapy costs per person were £87.

### Qualitative process evaluation

Five participants, 6 case managers and 50 stakeholders (HR, management) contributed to the qualitative work. One of the main obstacles encountered when setting up and delivering this study was the difficulties our local collaborators experienced with promoting the study across their organizations. Despite best efforts, their inability to identify and recruit a sufficient number of eligible participants was largely due to the constraints imposed by traditional management practices that mandated adherence to strict sickness absence policy and OH referral requirements which discouraged early OH referral in most circumstances.

‘We might have read between the lines that it was a mental health, potential mental health condition but it didn't spell it out with the words that you wanted so often when we look at referrals, we use a bit of intuition...’.

Despite this, the case managers reported positive feedback from their training in terms of enhanced clinical knowledge and skills development, and it provided a valuable opportunity to enhance professional capabilities.

‘it was good and the information that they gave you to be able to do the motivational interviewing and go through the case-management and the problem solving, I mean it was all well delivered and able to be lifted and taken back to my practice with no difficulties’.

We found case managers needed to possess strong and adaptive interpersonal skills in order to encourage participant engagement and lead collaborative discussions, particularly when resistance is evident.

From the participants’ perspective, the intervention provided a supportive environment for them to discuss and work on the obstacles and facilitators to their RTW. Some described the therapeutic benefits they experienced when engaging in collaborative problem-solving with their case managers; this positive interaction appeared to strengthen the participants’ engagement with the workplace.

‘Actually talking things through with somebody that was there to listen and not judge or compare… I think they did that in a good way’.

Nevertheless, the qualitative feedback provided an insight into the inherent complexities involved in RTW decision-making and coordination, reinforcing the importance of adopting a flexible and creative approach to case-management intervention. The intervention also opened up wider communication pathways between OH case managers and GPs and between participants, their line managers and colleagues. A comprehensive description of the qualitative work is described in full elsewhere.^27^

### WP 4 manualized intervention and recommendations for future development work and design for main study

Although we consider the case-management intervention fit for purpose and acceptable to participants, we removed the peer support and the video-recording of the case manager training features due to no uptake. Because of the screening and recruitment challenges, we considered it as not feasible to recommend a large-scale effectiveness trial unless a new system to increase OH referral rates for staff off sick with a CMD could be shown to be effective. Following advice from our Human Resource Department, the study team agreed that a targeted identification and screening approach involving HR reviewing sickness absence data on a weekly basis to identify employees on sick leave with CMHD with follow-up provision of a participant information sheet would likely optimize the number of participants taking part in a future trial. This type of method has been used successfully previously.^28^^,^^29^

## Discussion

### Main findings of this study

We were able to successfully develop a bespoke work-orientated case-management intervention to facilitate the RTW of NHS staff on sick leave with a CMD and successfully developed and delivered a case manager training workshop to upskill experienced OH nurses. An encouraging finding was that most components of the intervention were deemed as acceptable and useful by participants and the trained case manager intervention was feasible to deliver within the UK NHS. The costs associated with developing the intervention and its supporting resources were modest relative to potential benefits.

### What is already known on this topic

Health care workers have a high rate of sickness absence due to CMDs and the longer a worker is off work with CMD, the less chance they have of RTW. Long periods of sickness absence due to CMDs can have wider implications for individuals, the health care sector and economy.

### What this study adds

Our results contribute to the evidence that work-orientated multi-domain case-management interventions to facilitate earlier RTW for employees on sick leave with CMD could be clinically effective and cost-effective to deliver.^12^ In particular, as highlighted in Cullen’s *et al*.’s recent systematic review, such interventions support worker rehabilitation because they focus on core interventional features, i.e. ‘health care provision’, ‘service coordination’, ‘work adjustments’ and ‘multi-domain’. In addition, our study showed that the case-management intervention could be implemented alongside existing OH clinical practice, provided longer consultation appointments are available. However, delivery of the intervention is dependent on staff being referred to OH early on in their period of sickness absence. A shortcoming was our inability to encourage early OH referrals for the majority of staff off work with CMDs. This reflected similar participant screening and recruitment challenges experienced in other research, highlighting the difficulties with recruiting mental health patients into studies.^15^^,^^30^ We consider it as essential to ensure senior management support prior to intervention delivery to ameliorate existing policy barriers. Nevertheless, while earlier studies testing the effectiveness of RTW case-management interventions often showed sub-optimal adherence by participants and case managers,^31^^,^^32^ our findings showed there was good engagement with, and adherence to, most components of the intervention.

We acknowledge that a quarter (25%) of participants did not contribute baseline data; this was taken into consideration when we assessed the acceptability, feasibility and completeness of our proposed data collection tools. Considering this, we recommended that a useful strategy to optimize the questionnaire response rate in a future trial would be to administer and collect these at the time of participant consent and at follow-up review consultations or by providing participants with the option of completing questionnaires online rather than relying on the postal delivery-and-return method.

In addition, we recognize that the capacity of workplaces to successfully support sick-listed employees with CMD to RTW (including when supporting RTW-orientated intervention delivery) relies on all stakeholders (from executive to staff-level) contributing to a healthy workplace culture, which ultimately supports and underpins staff mental health and well-being. Although there is additional resource and financial implications for providing enhanced OH case-management support,^15^ we argue that any increase in costs could be offset if staff RTW sooner. However, the clinical effectiveness and cost-effectiveness would need to be assessed in a future trial. We found that our ‘mixed intervention/care as usual’ sites presented logistical challenges for local case managers and their colleagues to navigate in order to avoid any potential contamination.

Despite the challenges, the current research included an updated systematic review of the literature and the first national survey of OH services in the NHS regarding usual care for staff off sick with a CMD. We incorporated advice from our expert stakeholders (including patient representatives) throughout key stages of the study.

### Limitations of this study

The low number of eligible staff off sick with a CMD who were screened and recruited into the study, despite our best efforts to promote the study across management networks and implementation of an expanded screening method. The evaluation of the intervention could be tested by a stepped wedge design as this would allow researchers to test the new recruitment method in one organization with clear stop/go criteria before rolling out the intervention more widely. A stepped wedge design would also take account of temporal changes in sickness absence and cultural change. Alternatively, the case-management intervention could be trialled and tested in an individual randomized controlled trial.

## Data availability

De-identified datasets generated during and/or analysed during the current study are available from the corresponding author on reasonable request.

## Author’s contributions

All authors contributed to the study conception and design. Material preparation, data collection and analysis were performed by IM, VP, GN, DC, JS, MH, RS, PM, GG and DJ. The first draft of the manuscript was written by VP and IM, and all authors commented on subsequent versions of the manuscript. All authors read and approved the final manuscript.

## Notes

**V. Parsons**, PhD, MHM, MPH, BSc (Hons), Senior research fellow and research manager

**D. Juszczyk**, PhD, MSc, BSc, Post-doctoral research fellow

**G. Gilworth**, PhD, MPhil, MCSP, Ad Cert OH, Senior research fellow

**G. Ntani**, PhD, MSc, Diploma, Senior research fellow in epidemiology

**M. Henderson**, MBBS, MSc, PhD, MRCP, MRCPsych, Consultant Liaison Psychiatrist

**J. Smedley**, BM, BMed Sci, FRCP, Consultant occupational physician and senior lecturer

**P. McCrone**, PhD, MSc, BA (Hons), BSc (Hons), Professor of Health Economics

## Notes

**S. L. Hatch**, PhD, MA, BA, Social epidemiologist and senior fellow

**R. Shannon**, BSc, MSc, PhD, Lecturer and chartered psychologist

**D. Coggon**, MA, PhD, DM, FRCP, FFOM, FFPH, FMedSci, Emeritus Professor of Occupational and Environmental Medicine

**M. Molokhia**, PhD, MBChB, BSc, Clinical Reader in Epidemiology & Primary Care

**A. Griffiths**, PhD, MSc, BA, PGCE, PhD, CPsychol AFBPsS FFOM (Hon) FAcSS, Emeritus Professor of Occupational Health Psychology

**K. Walker-Bone**, BM, FRCP, PhD, Hon FFOM, Professor of Occupational Rheumatology

**I. Madan**, MBBS (Hons) MD FRCP FFOM, Professor of Occupational Medicine
